# Dealing with dilemmas in daily clinical practice: The development and evaluation of the seminar “suddenly, at the hospital” aimed at promoting professional identity formation in the final practical year of undergraduate medical education

**DOI:** 10.3205/zma001823

**Published:** 2026-03-23

**Authors:** Kristina Schick, Verena Kantenwein, Moritz Schumm, Teresa Bertram, Marie-Christine Fritzsche, Nikolaos Sapoutzis, Christopher Holzmann-Littig, Marjo Wijnen-Meijer

**Affiliations:** 1 TUD Dresden University of Technology, Medical Faculty and University Hospital Carl Gustav Carus, Institute of Medical Education, Dresden, Germany; 2 TUM Medical Education Center, Department Clinical Medicine, TUM School of Medicine and Health, Technical University of Munich, Munich, Germany; 3 Technical University Munich, TUM Klinikum Deutsches Herzzentrum, Department of Cardiology, Munich, Germany; 4 Technical University of Munich, Klinikum rechts der Isar, Department of Psychiatry, Munich, Germany; 5 TUM School of Medicine and Health, Technical University of Munich, Institute of History and Ethics in Medicine, Munich, Germany; 6 Technical University of Munich, School of Social Sciences and Technology, Department of Science, Technology and Society (STS), Munich, Germany; 7 Gesundheitsamt Hochtaunuskreis, Bad Homburg vor der Höhe, Germany; 8 Nephrocare Friedberg GmbH, Friedberg, Germany

**Keywords:** case-based clinical reasoning, storification, professional identity formation, medical professionalism, practical year, teaching development

## Abstract

**Aim:**

In everyday clinical practice, (future) physicians are confronted with dilemmas for which there are no clear solutions and which cannot be solved using clinical knowledge alone. To prepare medical students for such situations and support their formation of a professional identity, we developed a seminar that didactically applies elements of case-based clinical reasoning and storification to situations and potential professional strategies (medical professionalism).

**Project description:**

An interdisciplinary working group generated 28 ideas for cases depicting clinical dilemmas. These case ideas were assigned to five categories: Patient, colleague, supervisor, relatives, and self. These case ideas were then presented to medical students. A course concept was developed for the case idea in each category that garnered the highest level of interest. To develop the seminar sessions, teaching strategies were selected which encouraged reflection and participation. The course evaluation was carried out using a questionnaire.

**Results:**

The five seminar topics dealt with (1) no medical insurance (patient), (2) covering up a complication in medical treatment (colleague), (3) medical confidentiality (relatives), (4) conflict of roles (supervisor), and (5) romantic feelings toward patients (self). The seminar entailed five sessions, each one consisting of four teaching units. The course evaluations (N=16 practical-year (PY) students; age: M=26.50; SD=2.83; female: n=13) registered a high level of satisfaction among the students in regard to the course content and pedagogical approach.

**Conclusion:**

It was possible to successfully design and implement a course concept interlinking case-based clinical reasoning, storification and medical professionalism. The seminar enabled students to get a grasp on situations that go beyond clinical knowledge and develop strategies.

## Zusammenfassung

**Zielsetzung:**

(Angehende) Ärzt:innen sind im klinischen Alltag mit Dilemmata konfrontiert, für die es keine eindeutige Lösung gibt und die nicht ausschließlich mit klinischem Wissen gelöst werden können. Um Medizinstudierende auf solche Situationen vorzubereiten und die professionelle Identitätsentwicklung zu unterstützen, haben wir ein Seminar entwickelt, das Elemente der didaktischen Konzepte „Case-based clinical reasoning“ und „Storification“ auf Situationen und mögliche professionelle Strategien anwendet (medical professionalism).

**Projektbeschreibung:**

Eine interdisziplinäre Arbeitsgruppe entwickelte 28 Fallideen für klinische Dilemmata. Diese Fallideen bildeten fünf Kategorien ab: Patient:in, Kolleg:in, Chef:in, Angehörige und Selbst. Medizinstudierenden wurden die Fallideen zur Auswahl vorgelegt. Für die Fallidee pro Kategorie mit der höchsten Zustimmung wurde ein Kurskonzept ausgearbeitet. Für die Kursentwicklung wurden didaktische Konzepte ausgewählt, die zur Reflexion und Partizipation anregten. Die Kursevaluation erfolgte mittels Fragebogen.

**Ergebnisse:**

Die fünf Kurskonzepte befassten sich mit (1) dem Umgang einer fehlenden Krankenversicherung (Patient:in), (2) der Vertuschung eines Behandlungsfehlers (Kolleg:in), (3) der Schweigepflicht (Angehörige), (4) Rollenkonflikten (Chef:in), und (5) romantischen Gefühlen gegenüber Patient:innen (Selbst). Das Seminar umfasst fünf Termine mit jeweils vier Unterrichtseinheiten. Die Kursevaluation (N=16 PJ-Studierende; Alter: M=26,50; SD=2,83; weiblich: n=13) zeigte eine hohe Zufriedenheit der Studierenden mit dem inhaltlichen und didaktischen Konzept.

**Schlussfolgerung:**

Es konnte erfolgreich ein Kurskonzept entwickelt und implementiert werden, das „case-based clinical reasoning“, „storification“ und „medical professionalism“ miteinander verbindet. Das Seminar ermöglicht den Studierenden mit Situationen, deren Handhabung über das klinische Wissen hinausgeht, umzugehen und Handlungsstrategien zu entwickeln.

## Introduction

The demands placed on the role of the (future) physician are many-layered and complex: professional conduct is expected of them that includes, for example, respectful interaction with patients as individuals with consideration for their autonomy and wellbeing [1], [2]. In addition, physicians see themselves confronted with questions of fairness – as in structural injustices and unequal distribution – and issues regarding sustainability and economic responsibility [3], [4]. The *Marburger Bund Monitor 2024* points out that medical residents report a discrepancy between the daily work routine and their beliefs and values [5]. This finding indicates that young physicians are still not sufficiently prepared during medical school for the challenges of daily clinical practice [6].

The final practical year (PY) of medical school in Germany provides an opportunity to train intensively for complex procedures in clinical practice and develop one’s own attitudes toward it. The objective of the PY is to place students in a position to apply theoretical knowledge in daily clinical practice^1^. While the main focus of the current medical curricula in German-speaking countries is increasingly on teaching evidence-based medicine, it is nonetheless necessary that various dimensions – be they ethical, social, cultural or legal – are considered collectively and examined on a case-by-case basis when making decisions in everyday medical practice [3], [4]. The PY can be viewed as a liminal phase during which part of the future doctor’s professional development occurs. PY students do not belong entirely to the student body nor to the community of medical practitioners [7]. In the liminality of the PY, professionalism is imparted through positive and less positive role models [3]. This happens more in the form of narratives than as a result of clearly defined rules [3]. The freedom and unstructuredness of the liminality during the PY can encourage reflection and hence professional identity formation (PIF), but also hinder them at the same time [7].

Various approaches to defining PIF are described in the literature. The best-known definition was posited by Merton [8] and describes it as “thinking, acting and feeling like a physician” (p. 7). An initial definition in German elaborating on the meaning, explanation and goal of PIF was drafted by the DACH Association for Medical Education (GMA) PIF Working Group. Schick et al. [9] define PIF as an ongoing process of professional education and practice. This consists of the interaction between an individual and the surrounding environment and takes place consciously and unconsciously. Internal and external influences condition the development of knowledge, skills and a (self-)reflective attitude [9].

Up to now seminars in narrative-based medicine have been used to foster aspects of PIF. The transfer of medical topics to works of art (paintings, literature) is intended to enable students to reflect not only on medical topics, but also on less tangible topics such as patient relationships, collegial collaboration, and their own role as physician [10]. Group discussions, in-depth lectures and opportunities for creative reflection are often used as pedagogical techniques for delving more deeply into these topics [10].

Combining elements of case-based clinical reasoning (CBCR) and storification, our seminar follows a different teaching approach to foster PIF in the PY. CBCR is a proven educational concept for seminars to foster clinical reasoning [11]. In these seminars – often led by students – clinical cases are systematically worked on in small groups to demonstrate the pathways of medical decision-making and differential diagnostics. Whether the concept of CBCR can also be applied to solving dilemmas in daily clinical practice has not yet been investigated. One promising pedagogical approach for conveying the dilemmas in cases is “storification” [12]. Storification integrates curricular content into a narrative to put the learning material into a context and underscore its relevance [12], [13]. Studies have shown that storification increases students’ attention spans and, as a result, makes the teaching more engaging and effective [12], [14]. By combining elements of CBCR with storification, our seminar can focus on facing uncertainties [15]. Professional conduct can be negatively impacted by uncertainty, for instance, in regard to therapeutic regimens, different courses of disease, behaviors on a team, or in relation to patients and their families [16].

To reduce these uncertainties and foster professional conduct, the seminar pursues two main learning objectives:

1. By the end of the seminar, the students should be able to identify potential dilemmas in daily clinical practice and
2. reflect on their behavior and internal negotiation processes.

## Project description

To foster and promote the process of professionalization and PIF in future physicians, along with an integral discussion of topics in medical practice that go beyond medical expertise, a seminar project, “suddenly, at the hospital: Challenges in daily clinical practice”, was developed at the TUM School of Medicine & Health for PY students. Our seminar gives PY students an opportunity to explore various dilemmas that they may face later in everyday clinical practice and for which there are no predefined solutions. The principles of CBCR [11] and storification [12] were adapted for seminar implementation to depict practical, relevant and authentic daily situations, which will very likely arise in the future to confront the future physicians. These cases contain a wealth of information with aspects that need to be considered from different angles. The students discuss the different options for dealing with the dilemmas. The teachers serve more as “advisors” or “moderators” than as “instructors” and are available to answer questions. In the seminar, through the combination of CBCR [11] and storification [12], we aim to show students professional behavior that they can apply to different situations for the purpose of PIF while taking into account their own attitudes and positions [17]. No learning objectives are stated at any of the seminar sessions; instead the PY students are given opportunities to reflect in response to dilemmas based on case vignettes which can then be reflected on and discussed as a group, supported by an interdisciplinary team of moderators.

We brought together an interdisciplinary team in order to gather up holistic ideas during seminar development and include a wide variety of different perspectives. Seminar development and piloting were funded as part of the 2022 excellence strategy for the “pilot phase of project-based teaching and learning” at the Technical University of Munich (TUM). In the following, we present the project team and describe the process of seminar development and course evaluation.

### Project team

The project team was comprised of physicians from different specialties (nephrology (CHL), cardiology (VK), psychiatry (TB)), a representative from the humanities (MS), a medical ethicist (MCF) and a healthcare lawyer (NS), as well as two education scientists specialized in medical education (MWM & KS). The seminar design is based on the concept of interdisciplinarity to ensure a holistic development of the course format. Previous research has shown that the development of curricula covering the topics of professionalism and questions of socialization can benefit from the inclusion of specialists in education science, the humanities and other social sciences [18].

### Seminar development

The seminar was developed in several sequential steps:

#### 1. Development of the case ideas

A total of 28 scenarios with brief descriptions (case ideas) were developed through brainstorming by the participating physicians. Following this, the scenarios were reviewed by the development team, and commonalities between the scenarios were identified. Based on this, five clusters were inductively formed: Patient, colleague (team), supervisor (power), relatives, and self.

#### 2. Selection of the case ideas

In an initial pre-selection, the case ideas were discussed by the project team, and the team members voted for one case idea per cluster. To include the interests of the medical students in the selection of cases, we also put the 28 case ideas to a vote among 16 medical students, who were associated with the TUM Medical Education Center as doctoral candidates or student employees. The medical students could vote for one idea from each of the five main categories by answering the question, “Which case would you like to work on in a course setting?” For each cluster the case idea with the most votes was then selected. The responses by the students and project members were counted equally; the survey results were clear for all five clusters.

#### 3. Development of the teaching strategies

Groups of two to three project members developed each of the selected case ideas as a pedagogical concept for one seminar session. Various teaching methods were used to design the seminar sessions (see attachment 1 ). The sequence of events for the seminar session on “no medical insurance (patient)” is described as an example in the attachment 2 . Using these methods, the students should be able view a problem or issue from multiple perspectives and critically examine it. Students should be encouraged to reflect, and the fact that there is no single solution should be made clear to them.

#### 4. Recruitment of students and teachers

The students were informed about the seminar via MediTUM, the degree program management system, by sending multiple emails to PY students via mailing lists, and through posters and flyers. Likewise, the PY students working in the departments of the participating physicians were addressed directly and invited to participate. Registration took place via email.

### Course evaluation

The course evaluation was carried out using the evaluation form required by the funding source, “Projektbasiertes Lehren und Lernen an der TUM” (Project-based teaching and learning at TUM) and supplemented with specific questions. The required TUM evaluation form, developed by the funding provider, consisted of three open-ended questions and seven closed questions with rating scales (labeled end points: 1=completely dissatisfied, 5=very satisfied). The open-ended questions were

1. “What did you like best about the course?”
2. “How could the course be improved? Which expectations were not met?”
3. “What other methods could be used to support students during the project weeks/in the teamwork?”

The free-text responses were clustered by topic using MaxQDA Version 24 [19]. The seven closed questions elicited information regarding “satisfaction with seminar topics”, “support received”, “cooperation with teachers/peers”, “learning success regarding subject-based competencies”, “learning success regarding cross-sectional competencies”, “satisfaction with seminar results”, and “satisfaction with course organisation and length”.

The required evaluation form was supplemented to gather demographic information (age, sex, semester level, seminars already attended on topics in narrative medicine or self-development, vocational training, desired career/specialty, PY trimester, current PY specialty) and evaluate the individual seminar sessions using a 5-point Likert scale ranging from “completely irrelevant” (1) to “very relevant” (5) (labeled end points). The evaluation of the individual seminar days served to analyze the relevance of each topic for the students. The analysis of the scaled items was carried out descriptively with mean values and standard deviations using R Version 4.2.3 [20].

## Results

### Choice of topics

By means of an anonymous online survey held in the 2022/23 winter semester, the TUM medical students selected topics from the 28 case ideas divided according to the five clusters of Patient, Colleague (team), Supervisor (power), Relatives, and Self. Participating in this online survey were 16 medical students (68.8% female, age: *M*=23.63 years (*SD*=1.89)). The students were between the third and fifteenth semester of study (mode: seventh semester).

The following five cases were selected based on the survey:

- Patient: Admission of a young male patient without a residency permit (56.3%)
- Colleague: Covering up a complication due to medical treatment (50.0%)
- Supervisor: Taking on another shift in the ER without overtime compensation (62.5%)
- Relatives: Father is given a terminal diagnosis and does not want to tell his family (37.5%)
- Self: Physician develops feelings for a patient (50.0%)

### Design of the seminar topics

The five course topics with their dilemmas and the teaching methods are presented in attachment 1 . The seminar was taught by two instructors per seminar session; for several individual sessions (patient, colleague, relatives), an attorney specialized in healthcare law was connected via the internet for around an hour. The workload for the instructors amounted to approximately five hours per session (three full hours: seminar conduction; two full hours: preparation). Each session was taught by a medical teacher and a non-medical teacher (film studies, education science). The multidisciplinary tandem teaching sought to ensure that the medical aspects and experiences were covered and that the possibilities for reflection during the seminar were pointed out and opened up by the non-medical instructors.

The seminar was first introduced in the 2022/23 winter semester. A total of 19 PY students (age: *M*=26.72; *SD*=2.8; female: *n*=16) participated. Only four of the participants had previously attended a course in the medical humanities, narrative medicine or a comparable subject.

### Seminar evaluation

Overall, 19 students attended the seminar in the 2022/23 winter semester. At the end of the seminar during the last session, the participants who were present (*N*=16, age: *M*=26.5, *SD*=2.83 years; sex: female *n*=13) were asked to fill out a course evaluation. The participants were satisfied or very satisfied with the seminar in regard to all of the evaluation items (see table 1 (Tab. 1)). They expressed a high level of satisfaction with the choice of topics (*M*=4.62; *SD*=0.50) and cooperation with the instructors (*M*=4.94; *SD*=0.25). Satisfaction was lowest with the support provided by the university (*M*=4.06; *SD*=0.93) and their self-assessed gain in cross-sectional competencies (*M*=4.00; *SD*=0.63). 

Students were asked in open-ended questions

1. what they had really liked,
2. where they saw potential for improvement, and
3. what other methods could be applied to support the students.

#### 1. What did the students really like?

**Figure 1:**
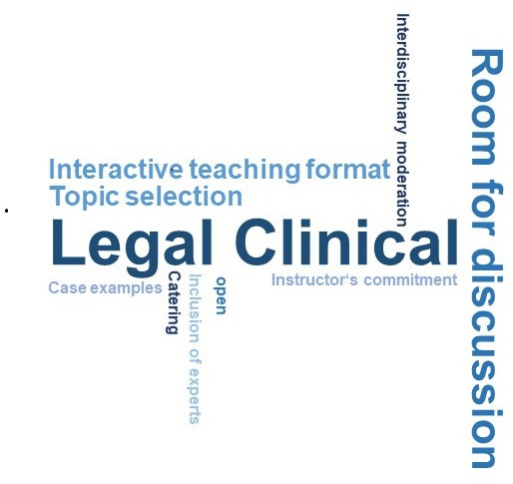
Word cloud for the question, “What did you like best about the course?” (number of mentions ≥3, participants: *N*=16)

The students had particularly enjoyed having plenty of room for discussion and interaction (see figure 1 (Fig. 1)). The students experienced this space as open and respectful. They emphasized the commitment of the teachers as well as the diversity of their expertise. The legal input from the healthcare lawyer was mentioned especially frequently (“legal clinic”). The students found it very helpful in terms of their future careers to receive some legal advice on the seminar topics. The mix of different teaching methods was viewed as diversified. The educational content was perceived as true to life and “not dry” as a result of including specific case examples.

#### 2. What could be improved?

The students desired concrete recommendations and strategies for how they could act in specific situations. The desire was expressed for summaries of the legal aspects. Also, a lecture was given during one of the seminar sessions, and the students did not find this type of frontal teaching motivating. They would have preferred to receive a written summary in advance and then using the classroom time for discussion and debate instead.

#### 3. What other methods would be valuable to support the students?

Making the materials and literature available via email in an easily accessible format was seen as a meaningful improvement going forward. Doing this on TUM's learning management system Moodle was ruled out by the PY students who were not enrolled at TUM. This obstacle must be avoided.

### Evaluation of the seminar topics and other requested topics

At the end of the course evaluation, the 16 participants were asked to rate the seminar topics and make requests for topics. The evaluation of the seminar topics is presented in attachment 1 (1=not at all relevant, 5=very relevant). The students identified “medical confidentiality” (*M*=4.73; *SD*=0.50) and “work/life (im)balance” (*M*=4.73; *SD*=0.59) as the most relevant topics and “romantic love in the hospital” as the least relevant (*M*=4.12; *SD*=1.02). The following issues were mentioned as other requested topics (at least two mentions): dealing with sexism, racism and managing relationships between the different healthcare professions or positions. Handling difficulties between colleagues and clearly defined expectations for the PY were considered to be relevant topics. Gender medicine was likewise a topic that the students would very much like to explore. Conflicts between paternalism and shared decision-making, as well as between morals and profits, meaning the conflict between billing optimization and “real”, medically meaningful care, were mentioned. Another thematic block entailed promoting resilience, supporting mental health in the medical professions (e.g., Balint groups during the PY already), and how to deal with wrong decisions and their consequences.

## Discussion

This project report describes the implementation of a seminar on the topics of professionalization and PIF during the practical year of medical study [21]. Adapting the teaching strategies of CBCR and storification to the topics of professionalization and identity formation enabled the PY students to work on issues in daily clinical practice that go beyond medical expertise. The interdisciplinary composition of the project team allowed us to design the seminar topics to encompass multiple points of view. The courses of action open to physicians were thus communicated to the students, and also enhanced and supplemented with methods and perspectives of non-physicians [18].

As a result of its interdisciplinary design, the seminar was an opportunity to make the hidden curriculum partially visible in the PY. Allit und Frampton [15] assume that the hidden curriculum imparts behaviors, values and characteristics which (future) physicians (should) accept and adopt [15]. These behaviors can be both conducive and destructive. Alongside positive models of conduct, behavior patterns are also imparted which supervisors often do not wish to pass on, such as cynicism, fear of legal consequences, and rigid hierarchical structures [22], [23]. By engaging with topics such as a collegial collaboration, handling errors (in a team) and medical confidentiality, the implicit expectations of the medical community and other stakeholders can be made visible. Likewise, the (future) physicians’ ability to reflect can also be fostered as a result, so that they can identify both constructive and destructive behaviors and determine how they will respond to them.

Our selection of cases reflect six domains, which are also described in the six domains according to Hilton & Slotnick [24]: personal traits such as reflection and self-awareness, ethical practice, responsibility and accountability (critical thinking, life-long learning, striving for excellence), and cooperative traits such as respect for patients, teamwork and social responsibility [3], [24]. The positive evaluation of the seminar by the students also showed that there is a need in undergraduate medical education to address these topics and offer space for reflection during medical study and, above all, to support the building up of resilience [25], [26]. Although the required courses taken during the clinical phase of medical school do in part cover these topics – such as the course on history, theory and ethics in medicine – ongoing and structured opportunities for in-depth reflection on these topics are still missing during the PY. The topics handled up to now focus on the five clusters, self, patient, colleague, relatives, and supervisor, and thus provide direct personal relevance. Another important thematic complex is the healthcare system itself which implicitly resonates in many of the clusters named above, not only in the case involving the uninsured patient, but also in the one addressing the work/life imbalance. A case explicitly dealing with the system-specific challenges could be considered during further development of the seminar.

One special feature of the seminar is that students learn, or are supposed to learn, how to deal with uncertainty and ambiguity. The cases were designed such that they have no clear answer, but rather can be approached from a variety of angles. However, these different approaches can in part stand in opposition to each other. Becoming aware of and dealing with these oppositions was the seminar's focus – as a contrast to the guideline-oriented and algorithmically informed undergraduate medical curriculum.

Nonetheless, the extent to which the seminar enables PY students to apply their knowledge to their daily conduct or actions remains an open question. One challenge is to teach these topics in such a way that avoids superficial behavior. Superficial behavior can manifest when the students only imitate the supposedly desired behaviors in order to appear professional [27], [28]. “Professionalism” is often perceived as an abstract construct that seems mostly intangible [3], making the desire for concrete, recommended actions, as was mentioned in the course evaluation, a plausible one. At the same time, this desire to receive such recommendations shows us that the real idea behind the seminar – to grapple and come to grips with contradiction and uncertainty – is not yet fully implemented. In order to further develop the seminar, it is necessary to explore the possibilities for reflection in more depth. The underlying theoretical content could be moved forward and taught in an e-learning unit thus creating space for reflective phases during the classroom-based sessions. Experiences could also be shared and discussed in more detail in the large and small groups. The requested topics also show that the future physicians are very concerned about resilience, mental health, and potential conflicts between roles. The other topics that were mentioned are already addressed, e.g., racism and gender medicine in “love in the hospital” (self), morals and profits in “no medical insurance” (patient). Hence these topics can easily be expanded on in the existing seminars.

Conducting the seminar entailed several challenges. A number of people with different academic backgrounds were involved which meant that project development required considerable resources and could only be implemented in the way that it was through additional funding. Recruiting the instructors, who were not members of the project team, had to be done carefully. They needed to be able to identify with the seminar topic and have an affinity for topics relating to PIF.

Each seminar session was designed to consist of four teaching units. The sessions were held in the afternoon and overlapped with the PY work schedule by an hour. The PY students had to be released from work to attend the seminar so as to avoid incurring any absences for their participation. This release from work was intended to motivate the students to participate in the seminar. However, this approach was connected with increased administrative effort since the PY coordinators at the hospitals and on the wards had to be informed and the students had to be released on time to attend the seminar. The original scope of the seminar, which had 20 teaching units, markedly expanded the PY coursework so that the amount of time will be reduced the next time the seminar is offered. In the future, it will be possible (at the TU Dresden) to attend the seminar sessions separately without an obligation to participate in all five sessions, thus enabling a larger number of PY students to benefit from the course. To extend the seminar offering to other PY students, the course concept can also be transferred easily to other interested medical schools. The teaching and learning materials are compiled in a Moodle course which can be made available to interested faculties.

It must be viewed as a limitation that seminar attendance was voluntary making it possible that primarily PIF-oriented students participated. The question arises as to what the evaluation would have been if the seminar had been required.

Our project also leaves the questions open regarding how the learning success can be assessed and if the seminar actually fosters professional conduct. However, in general there are still big challenges in how professionalism or PIF can be judged overall. To date, the focus has been on the ability to reflect and the description of narratives and less so on examining the actual professional conduct [28].

Another challenge is that, in order to hold the seminar, a medical and a non-medical individual should be recruited to teach it. This aspect involves more organizational effort, but should be retained to ensure the holistic approach of the seminar's concept. The inclusion of a healthcare attorney can be a challenge in terms of organization and availability, but will be viewed by the students as especially insightful.

## Conclusion

With our seminar “suddenly, at the hospital” it has been possible to offer topics on professionalism for PY students that go beyond purely clinical knowledge. To do this, we connected the teaching strategies of CBCR and storification with questions about topics such as handling confidentiality, legal aspects, and personal challenges. This combination enabled the students to reflect on possible options in discussions with their peers and the instructors. The positive student evaluation of the seminar shows that there is a need to impart this content during the PY. The initial development of the topics required effort and resources, but the seminar sessions themselves can now be easily implemented and transferred to other medical schools. The seminar is now offered as an optional course during the PY at TUM. Furthermore, this concept will be transferred, developed further and implemented at the Dresden University of Technology as a required component of the PY coursework. As a result, there will be opportunity to investigate in more detail the effectiveness of such a course concept.

## Note

^1^ In Germany, the PY comes after the second state medical exam and is the final year of undergraduate medical school (year 6). During this year, students rotate in 48 weeks through three trimesters in the hospital setting: one trimester of Internal Medicine, one trimester of Surgery, and one trimester of an elective subject. The third state medical exam that culminates in medical licensure is taken at the end of the PY.

## Authors’ ORCIDs

- Kristina Schick: [0000-0002-4819-4604]
- Verena Kantenwein: [0000-0003-1182-8228]
- Moritz Schumm: [0009-0008-2663-7815]
- Teresa Bertram: [0000-0002-5744-1531]
- Marie-Christine Fritzsche: [0000-0002-8056-2462]
- Christopher Holzmann-Littig: [0000-0002-9849-148X]
- Marjo Wijnen-Meijer: [0009-0006-4861-8098]

## Competing interests

The authors declare that they have no competing interests. 

## Supplementary Material

Overview of the course concept with case ideas, dilemmas and pedagogical approach to the cases, along with student evaluations of the individual cases (N=16)

Instructor guideline

## Figures and Tables

**Table 1:**
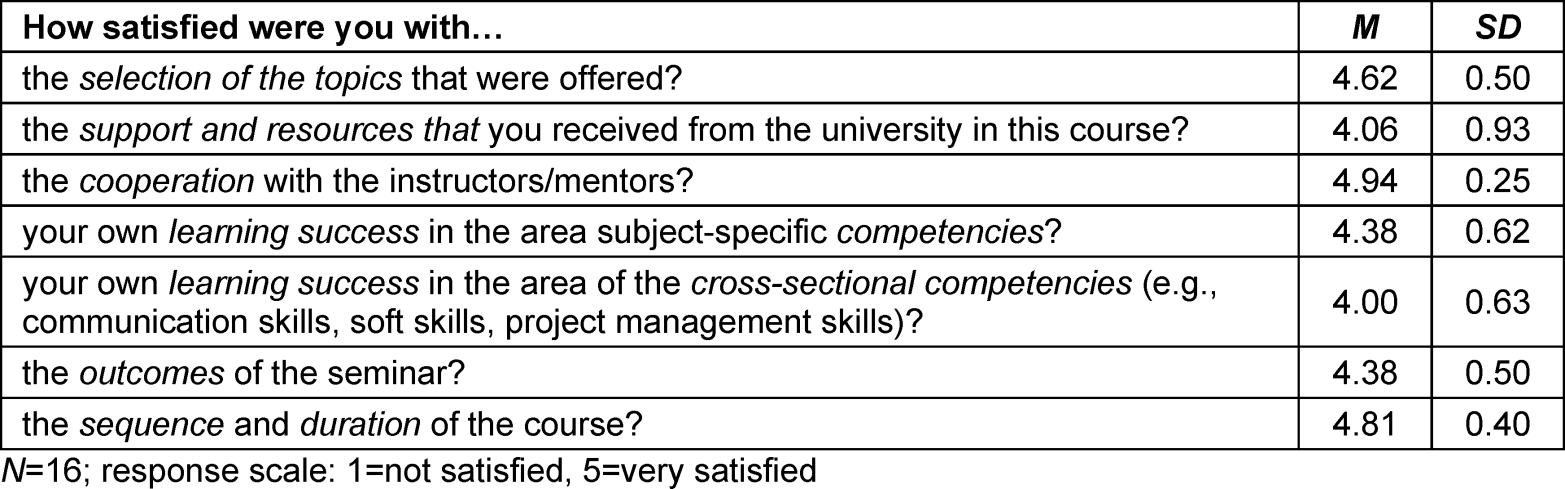
Results of the seminar evaluation
