# Supplementary material for: Dealing with dilemmas in daily clinical practice: The development and evaluation of the seminar “suddenly, at the hospital” aimed at promoting professional identity formation in the final practical year of undergraduate medical education
Source: GMS J Med Educ. 2026 Mar 23;43(3):Doc29. doi: 10.3205/zma001823 (PMC13054812; doi:10.3205/zma001823)
Supplement: Overview of the course concept with case ideas, dilemmas and pedagogical approach to the cases, along with student evaluations of the individual cases (N=16) [file JME-43-29-s-001.pdf]

Attachment 1: Overview of the course concept with case ideas, dilemmas and pedagogical approach to the cases, along with student evaluations of the individual cases (*N*=16)

Case idea	Dilemma	Teaching strategy	Evaluation [M(SD)]
"Is silence golden?" (Duty of confidentiality)			
A) A father of two small children receives a terminal diagnosis (glioblastoma). Over the course of several conversations with the patient, you discover that neither his wife nor his children have been told about the disease. The patient does not want to tell his family members anything and instead enjoy the time remaining. You know the wife well. What do you do? How do you handle this?<br>B) You are treating a male HIV patient with a high viral load who has not told his partner anything about it. Can or must you inform her?	• When is it permitted to break medical confidentiality?<br>• Conflict between the duty to remain silent and loyalty to friends/relatives<br>• When is there a health risk to a third person?	• Small-group assignment<br>• Formation of an ethics council to advise a person faced with a dilemma<br>• Input regarding legal context	4.81 (0.54)
"At sea and hearing voices" (No medical insurance)			
You admit a young male patient. It becomes evident that he is in Germany without a residency permit or health insurance. The admissions office tells you he can only be treated if it is an emergency which you must formally attest to. The patient is very sick and probably won't receive any care elsewhere but his condition is not life-threatening. What do you do?	Conflict between<br>• The basic medical understanding of wanting to help all patients<br>• Responsibility for allocating resources in the healthcare system<br>• Legal context (giving false testimony)<br>• Economic pressure (who will pay for the medical treatment?)	*Group discussion with three perspectives:*<br>• <u>Patient:</u> What can lead to someone not having any medical insurance? → Leave the stereotypes behind<br>• <u>Hospital management:</u> Economic pressures<br>• <u>Physician:</u> Caught between the oath that was sworn and being an employee	4.36 (0.93)

Attachment 1 to Schick K, Kantenwein V, Schumm M, Bertram T, Fritzsche MC, Sapoutzis N, Holzmann-Littig C, Wijnen-Meijer M. *Dealing with dilemmas in daily clinical practice: The development and evaluation of the seminar "suddenly, at the hospital" aimed at promoting professional identity formation in the final practical year of undergraduate medical education.* GMS J Med Educ. 2026;43(3):Doc29. DOI: 10.3205/zma001823

Case idea	Dilemma	Teaching strategy	Evaluation [M(SD)]
"Complicated consequences" (Concealing a complication due to treatment)			
The team hides a complication in the treatment of a patient.<br>What to do if you are one of the people involved?<br>What to do if you are not involved?	• Morals, ethics<br>• Lack of loyalty to colleagues, self-protection<br>• Legal situation<br>• Superiors' position of power	• Choose-your-own-adventure book<br>• Support from medical attorneys	4.73 (0.46)
Work/Life imbalance (Workload)			
Emergency department: You are asked to fill in for someone but you have a family event that has been planned long in advance and is very important to you. However, if you don't help out, the ER will be short staffed and adequate patient care won't be guaranteed. What's your decision?	• Lack of loyalty to colleagues<br>• Risk of burn-out<br>• Legal situation<br>• Superiors' position of power<br>• Systemic problems in post-graduate education	• Group discussions: each participant outlines their position with keywords and pins these up on pin boards after the discussion.<br>• Then a full group discussion with support from experts.	4.73 (0.59)
"A fateful encounter" (Love in the hospital)			
In your private life you met someone and you've come to know each other more closely. Several days later this person is admitted to your hospital ward. You are now supposed to carry out medical exams, such as blood and stool samples. How do you handle this? How can this private connection influence your professional conduct?	• (Implicit) bias in medicine<br>• Do we treat all patients equally? Patients who we like? Those who we don't like? Patients who are like us or familiar to us (educational background, language, cultural background, etc.)? Men and women?	• Photo love story, break-out session,<br>• Group discussion, break-out sessions with different materials, e.g., *Harvard online implicit bias test*,<br>• YouTube video, John Oliver: *Implicit bias in medicine*,<br>• Article: "*Implicit bias in healthcare professionals: a systematic review"*	4.12 (1.02)

The evaluation was carried out using a 5-point rating scale ranging from 1=completely irrelevant to 5=very relevant
