# Supplementary material for: Dealing with dilemmas in daily clinical practice: The development and evaluation of the seminar “suddenly, at the hospital” aimed at promoting professional identity formation in the final practical year of undergraduate medical education
Source: GMS J Med Educ. 2026 Mar 23;43(3):Doc29. doi: 10.3205/zma001823 (PMC13054812; doi:10.3205/zma001823)
Supplement: Instructor guideline [file JME-43-29-s-002.pdf]

# Attachment 2: Instructor guideline

| **Course/Seminar: "Case-based medical professional reasoning" – case 2 – no medical insurance**<br><br>**Case 2** focuses on how, as an attending physician, to deal with treating patients in socially difficult circumstances and shows how a lack of resources, economic pressure and legal factors influence a physician's room to maneuver. A patient's story is told as the input: A young man with psychotic symptoms comes to the ER. It is determined that he does not have valid residency status or healthcare insurance. He is severely ill and probably won't receive help elsewhere. According to hospital management, treatment can only be given if it is an emergency. Otherwise, the patient himself must pay for the treatment or the hospital will have to cover the costs. The students need to consider what they would do in this situation. Presumably there is no acute threat to his life at the moment. However, an imminent exacerbation of the disease in the absence of treatment could quite possibly lead to self-endangerment in the patient, e.g., in the form of urgent voices ordering suicide or a distorted perception of reality. | **Group composition:**<br>Medical students in the clinical phase of study and PY, students in nursing science and sports science<br><br>**Date and time:**<br><br>**Room:**<br><br>*Instructors:*<br>*1 male physician / 1 female physician*<br>*1 social scientist*<br>*1 healthcare attorney* |
|---|---|

| **Context and relevance** | **Learning objectives** |
|---|---|
| Case-based learning in undergraduate education often involves making diagnoses and imparting medical expertise. In daily clinical practice, however, the treatment of patients is also impacted by their social/cultural milieu, institutional forces such as increasing economic pressure or lack of resources, through the different demands placed on roles, hierarchies and dependencies, as well as legal factors. This seminar is meant to give students the opportunity to critically examine all of these influencing factors and consider their future role as physician. | The students should be able to identify and name reasons why a patient does not have medical insurance.<br>The students explore the legal and ethical principles that could play a role in the treatment of patients without healthcare coverage. |

Attachment 2 to Schick K, Kantenwein V, Schumm M, Bertram T, Fritzsche MC, Sapoutzis N, Holzmann-Littig C, Wijnen-Meijer M. *Dealing with dilemmas in daily clinical practice: The development and evaluation of the seminar "suddenly, at the hospital" aimed at promoting professional identity formation in the final practical year of undergraduate medical education.* GMS J Med Educ. 2026;43(3):Doc29. DOI: 10.3205/zma001823

| **Schedule** | **Content and activities** | **Pedagogical approach** | **Materials** | **Organization / participating instructors** |
|---|---|---|---|---|
| **Introduction**<br>*20 min.* | Arrival, words of welcome and introduction to the seminar by the instructors; introductory round<br>Questions for the introductory round, e.g.:<br>- Which semester level/PY subject?<br>- Why did you register for this course? | Large-group conversation | None | Students sit in a circle or semi-circle<br>(Physician/<br>social scientist) |
| **Case presentation**<br>*15 min.* | Students read the case description | Individual reading | Material 1: Printed case description<br>*Alternatives: Reading aloud, PowerPoint* | Students sit in a circle or semi-circle<br>(Physician/<br>social scientist) |
| **Group discussion I**<br>*25 min.* | Open discussion:<br>Guiding questions:<br>- What would you do in this situation?<br>- Which different interests and values come into conflict with each other here?<br>- Which problems could result from this situation? | Allow space for first impressions and emotional aspects; What decisions or actions would the students intuitively take in this situation without having much prior knowledge? | Record the impressions / statements on a flipchart or white board | Students sit in a circle or semi-circle<br>(Physician/<br>social scientist) |
| **Legal/ethical input**<br>*15 min.* | Presentation with around 5 slides on:<br>- Differentiating between law, morals and ethics<br>- Which legal aspects must be taken into account? (confidentiality, failure to render assistance, giving false testimony)<br>- Which ethical aspects must be considered, e.g. just distribution of resources, economic viability, doing good/doing no harm | Instructors present the slides | Laptop, projector and screen | Students sit in a circle or semi-circle<br>(Physician/<br>social scientist/<br>healthcare attorney) |
| **Break**<br>*10 min.* | | | | |

| Schedule | Content and activities | Pedagogical approach | Materials | Organization / participating instructors |
|---|---|---|---|---|
| **Group assignment**<br>*40 min.* | **Student are divided into three groups**<br><br>Perspective A: Patient – What can cause someone to not have medical insurance? Which stereotypes exist in society?<br><br>Perspective B: Hospital management – What does economic viability mean for a hospital?<br><br>Perspective C: The person giving medical care:<br>Which legal and ethical aspects influence the decision? | Each group takes a different perspective and is given the corresponding material<br><br>Divide into groups, e.g., counting off by 3s<br><br>Instructors distribute iPads and let the groups work independently | *Links to the materials via Moodle for each of the three points of view* | Students work in the room divided into three small groups<br>(Physician/<br>social scientist/<br>healthcare attorney) |
| **Group discussion II**<br>*45 min.* | **Presentation of the results and second round of discussion** | Large-group conversation,<br>Gather the contexts and discuss the various points of view,<br>Healthcare attorney classifies the decisions and comments on them | Record the key statements in the discussion on a flipchart or white board | Students sit in a circle or semi-circle<br>(Physician/<br>social scientist/<br>healthcare attorney) |
| **Conclusion**<br>*10 min.* | **Closure of the seminar session** | Lightning round<br>(What are your 3 takeaways from this session?) | None | ((Physician/<br>social scientist) |

Attachment 2 to Schick K, Kantenwein V, Schumm M, Bertram T, Fritzsche MC, Sapoutzis N, Holzmann-Littig C, Wijnen-Meijer M. *Dealing with dilemmas in daily clinical practice: The development and evaluation of the seminar “suddenly, at the hospital” aimed at promoting professional identity formation in the final practical year of undergraduate medical education.* GMS J Med Educ. 2026;43(3):Doc29. DOI: 10.3205/zma001823

**Required Materials:**

- iPads with Moodle access
- Printouts of the case description (one for each participant)
- Board/white board/flipchart/pinboard to record points made during discussion
- Laptop, projector
- Facilitator's toolbox

**Case Description**

It is November 27. You are called to a young patient in the emergency room; you estimate he is in his mid to late twenties. The admitting nurse is around 45 years old and has worked at this job for more than 20 years. Thanks to his experience, he is viewed as the unofficial boss on the shift and behaves like it too. He says, passersby had called an ambulance because the patient was speaking to himself in a loud voice and seemed confused. He hadn't been able to find out any more information because "the guy can't speak German." He points out to you that the patient does not have any identification on him. Looking at the health insurance card he voices doubt: The picture and the recorded details do not match the person in front of them. He assumes that it involves a patient without a residence permit or healthcare coverage "who wants to surreptitiously get medical care."

You speak to the patient in English. A thin leather jacket is lying on his lap. He swings his legs so that he keeps having to catch and reposition the jacket. The smell makes it evident that it has been awhile since the patient or his clothes were washed. He runs his hand frequently through his hair and looks around constantly. He is somewhat disoriented in terms of time and place (but knows the month and year, as well as the city, but not the weekday or the name of the hospital).

Two months ago he started working as a cook on a cruise ship. It was bad onboard the ship. The head chef and the co-workers treated him disrespectfully and ostracized him. He felt he was being constantly surveilled and developed a very uneasy feeling, as if something bad would happen soon. He was no longer able to sleep properly. And he had voices in his head. When asked say more in detail about the voices, he said: They could make him do things and control his emotions. They commented on what he did or insulted him. He talks with them to quiet them down. They say he is "a faggot" and should die, jump off the ship, for instance. He has known these voices before. In Tunisia he saw a doctor who said that he has schizophrenia, he has the medical report with him now. His uncle is also schizophrenic. The patient's hands are balled into fists as if under strain.

He is from Tunisia. He trained as a cook and learned to prepare Tunisian and French cuisine at *L'Astragale*; he's worked a lot and always enjoyed it. Cooking saved him. All the mental health problems in his youth receded into the background. Before that, after his parents divorced, he had

psychoses, heard voices in his head. Work, creativity, recognition on the team helped. Until 2011 when Ennahda came to power and restricted liberal lifestyles in Tunisia. He recounts the reprisals under which he suffered due to his open homosexuality. He was beaten up several times on the street. Forms of government had changed a lot in the time up to Saied, but people's way of thinking has been "poisoned." His disease showed up again, negatively affecting his work. Too many absences so he was let go. He is very happy to have gotten the job on the cruise ship. It finally got him out of Tunisia. But onboard was so bad for him that he jumped ship in Passau without saying a word to anyone. He would rather live in Germany anyway. He made it to Munich in a roundabout way. He has just been treated at the Isar-Amper-Klinikum Ost, where he was given olanzapine 20 mg/d, but was discharged after two days. He does not know why. He was not given any of the medication to take with him so he hasn't taken any for a week now. He continues to hear voices, but none that order him to commit suicide. He doesn't know what he should do. He can't go back to Tunisia. You mention the health insurance card to him, which causes him to sigh deeply while his eyes look up toward the ceiling. After a short pause, he admits that it is not his card. He also confirms in a near whisper that he has no valid visa. The visa is only valid for being on the cruise ship and since he has left the ship, he no longer has any medical insurance.

You speak again with the hospital admissions office, which does not want to admit the patient: "Where would we put him? And the hospital would be stuck with the costs!" He can only be treated if it is an emergency and you would attest to this. Otherwise, he must pay the costs himself. What do you do?
